# Supplementary material for: Autism trends in a medium size coastal town of England
Source: PLoS One. 2023 Jun 30;18(6):e0287808. doi: 10.1371/journal.pone.0287808 (PMC10313016; doi:10.1371/journal.pone.0287808)
Supplement: S1 File — S1 Fig Prevalence (per 1000 people) for autism in Fleetwood from 2002 to 2020; S2 Fig Incidence (per 1000 people) for autism in Fleetwood from 2002 to 2020; S3 Fig Prevalence (per 1000 people) for Asperger’s syndrome in Fleetwood from 2002 to 2020; S4 Fig Incidence (per 1000 people) for Asperger’s syndrome in Fleetwood from 2002 to 2020; S1 Table Contingency table for autism diagnoses up to 2020; S2 Table Contingency table for Asperger’s syndrome diagnoses up to 2020; S3 Table Annual chi-squared tests for gender between 2002–2020; S4 Table Coefficients summary for the logistic regression models for autism diagnoses; S5 Table Coefficients summary for the logistic regression models for Asperger’s syndrome diagnoses. (DOCX) [file pone.0287808.s001.docx]

**Autism trends in Fleetwood: Supplementary File**

**Supplementary figures**


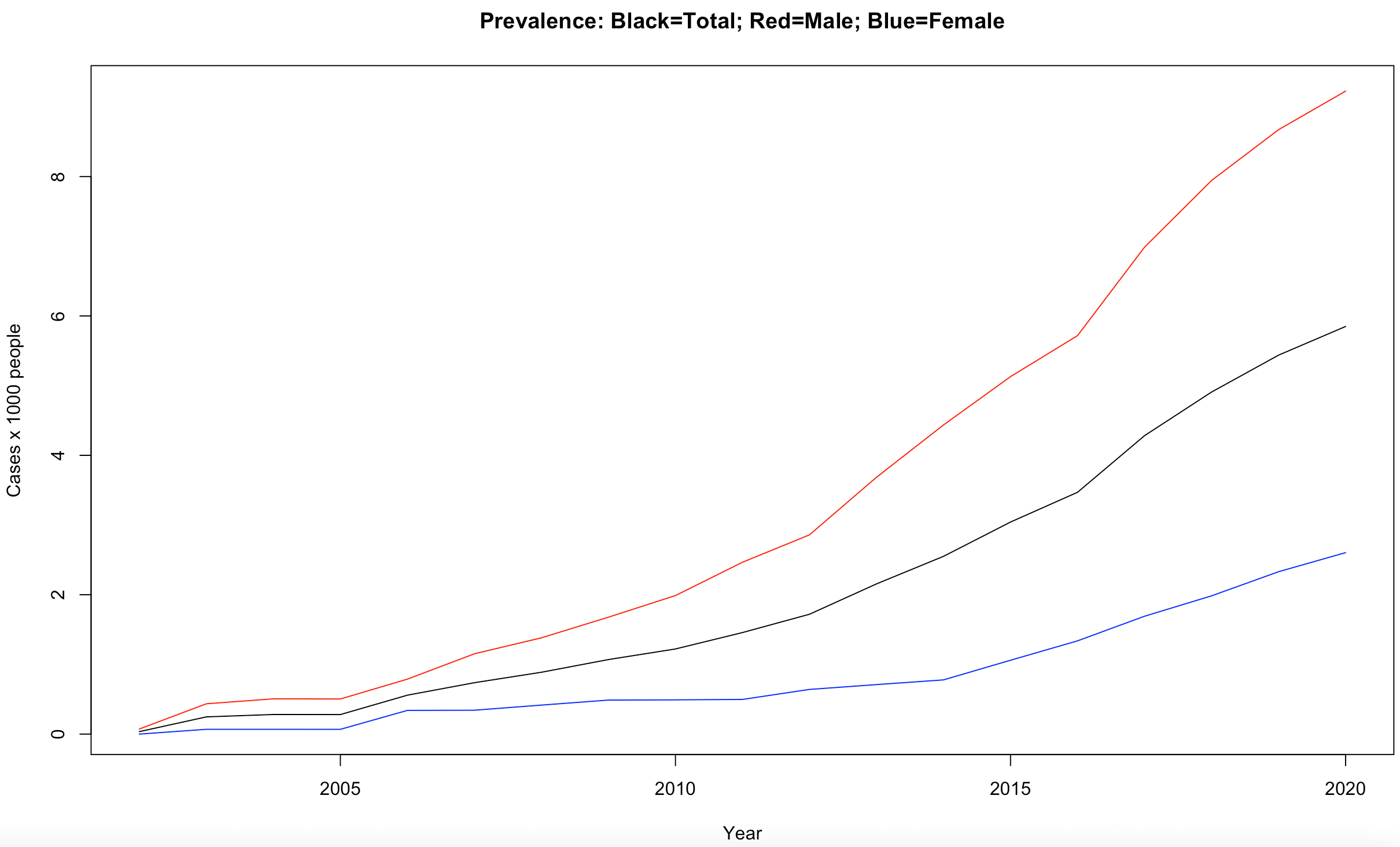


**S1 Fig. Prevalence (per 1000 people) for autism in Fleetwood from 2002 to 2020.** Lines legend: black = combined male and female autism prevalence, red = male autism prevalence, blue = female autism prevalence.


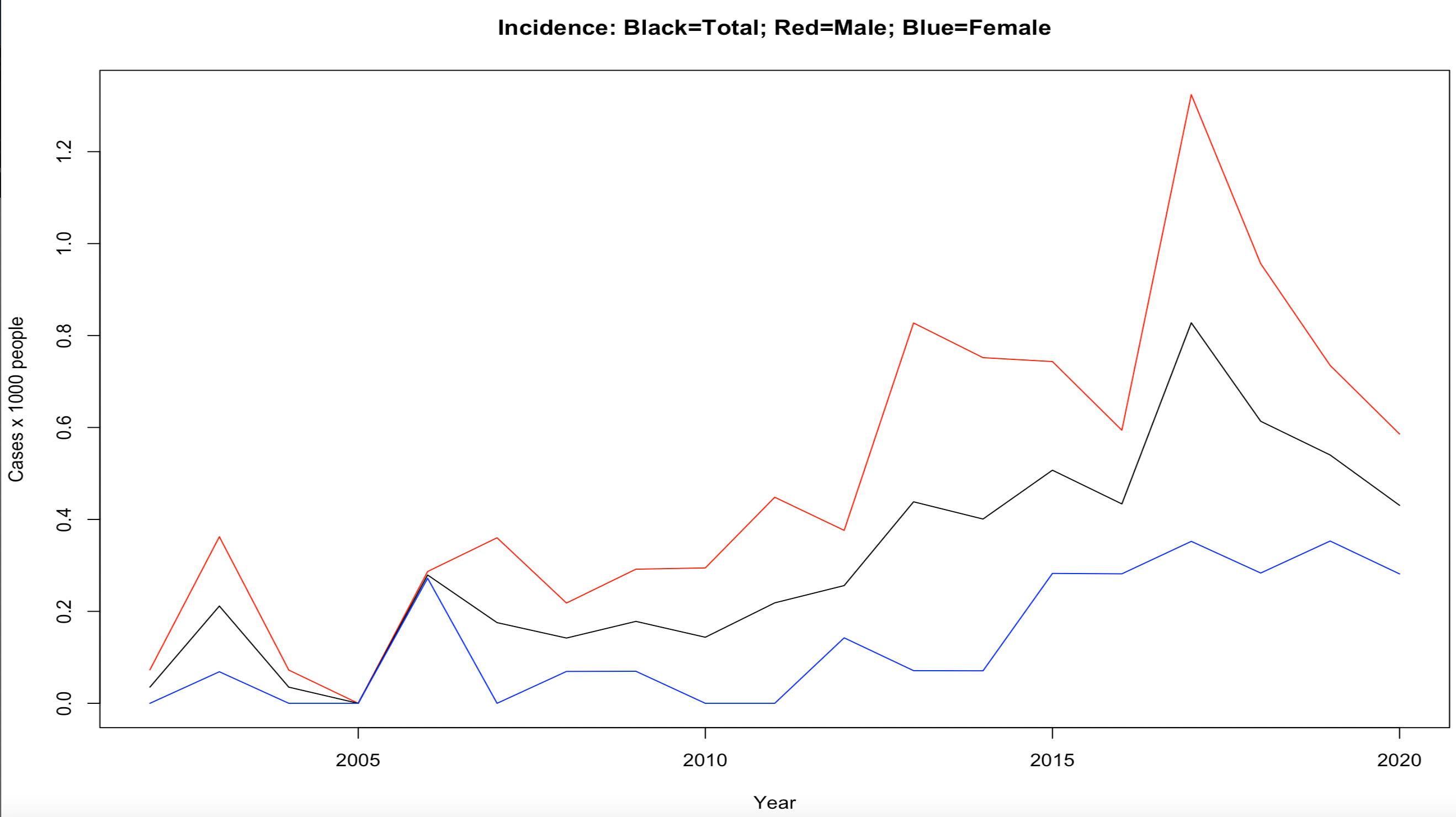


**S2 Fig. Incidence (per 1000 people) for autism in Fleetwood from 2002 to 2020.** Lines legend: black = combined male and female autism incidence, red = male autism incidence, blue = female autism incidence.


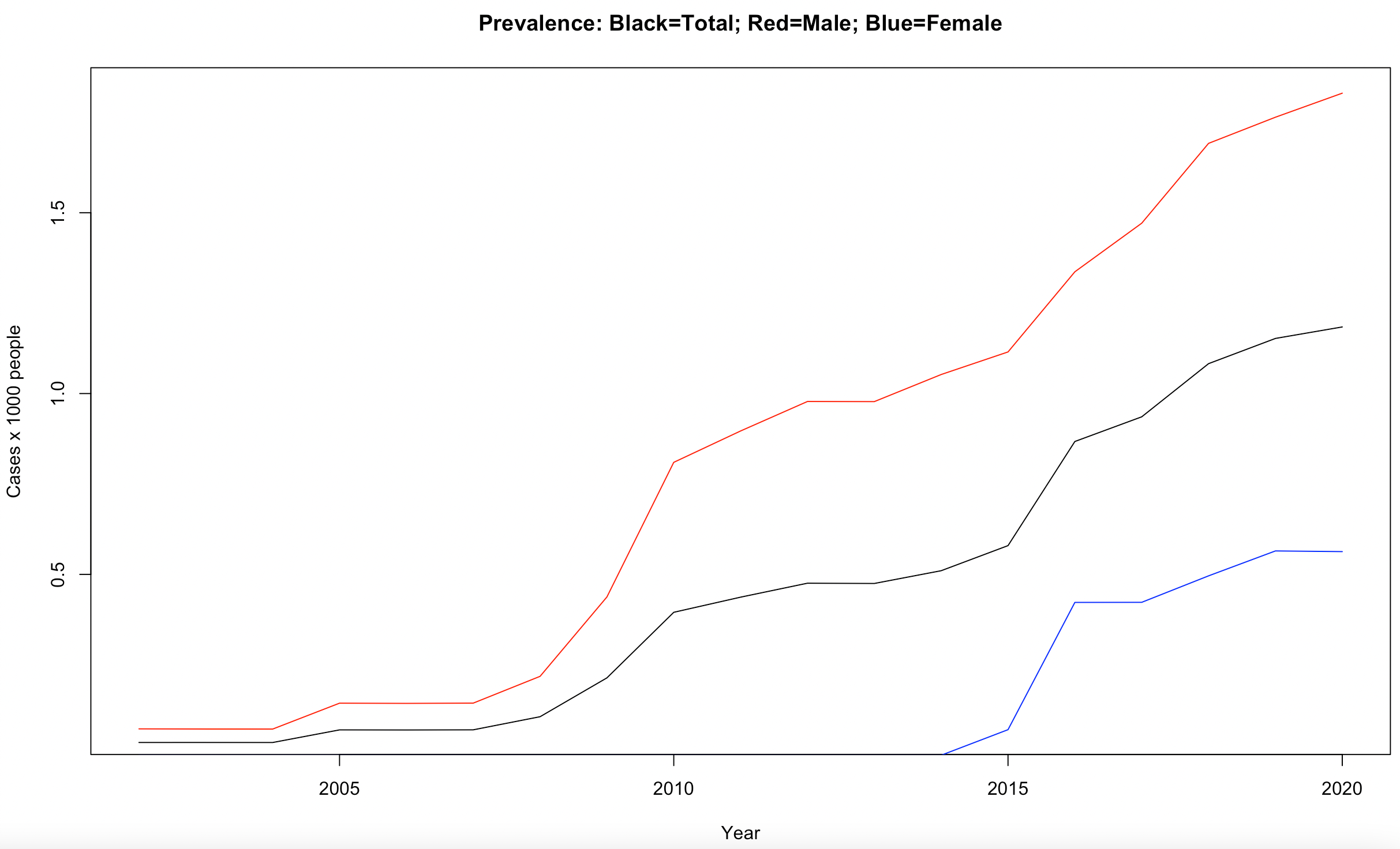


**S3 Fig. Prevalence (per 1000 people) for Asperger’s syndrome in Fleetwood from 2002 to 2020.** Lines legend: black = combined male and female Asperger’s syndrome prevalence, red = male Asperger’s syndrome prevalence, blue = female Asperger’s syndrome prevalence.


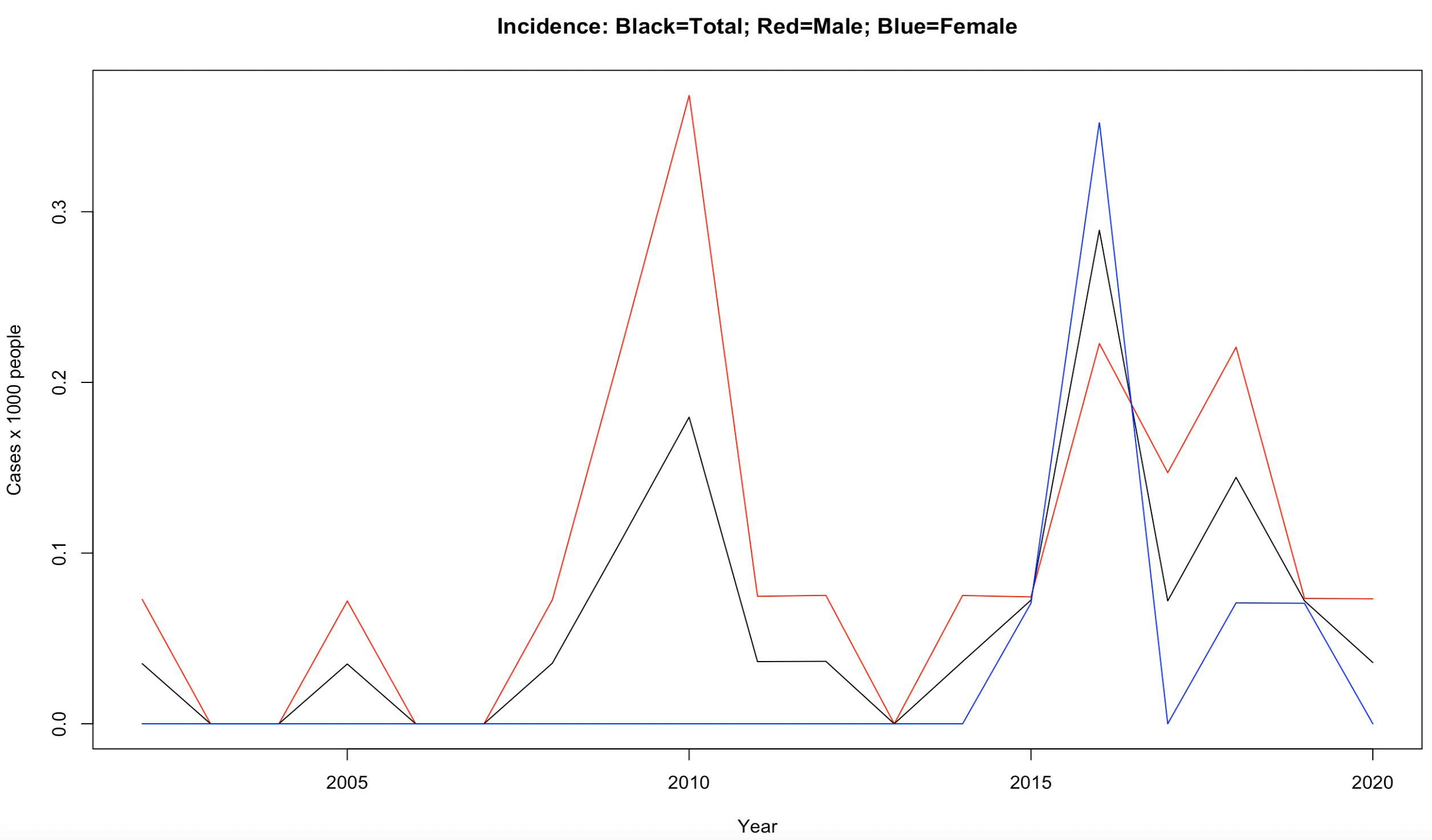


**S4 Fig. Incidence (per 1000 people) for Asperger’s syndrome in Fleetwood from 2002 to 2020.** Lines legend: black = combined male and female Asperger’s syndrome incidence, red = male Asperger’s syndrome incidence, blue = female Asperger’s syndrome incidence.

**Supplementary tables**

**S1 Table. Contingency table for autism diagnoses up to 2020.**

|  | Autism | No Autism |
| --- | --- | --- |
| Male | 126 | 13531 |
| Female | 37 | 14173 |

**S2 Table. Contingency table for Asperger’s syndrome diagnoses up to 2020.**

|  | Asperger’s syndrome | No Asperger’s syndrome |
| --- | --- | --- |
| Male | 25 | 13632 |
| Female | 8 | 14202 |

**S3 Table. Annual chi-squared tests for gender between 2002-2020.**

| Year | chi-squared value | p-value |
| --- | --- | --- |
| 2002 | 0.56932 | 0.4505 |
| 2003 | 1.6667 | 0.1967 |
| 2004 | 0.00077 | 0.9779 |
| 2005 | 0.00069439 | 0.979 |
| 2006 | 1.5632e-29 | 1 |
| 2007 | 3.4058 | 0.06497 |
| 2008 | 0.89846 | 0.3432 |
| 2009 | 3.3567 | 0.06693 |
| 2010 | 7.5076 | <0.05 |
| 2011 | 5.4528 | <0.05 |
| 2012 | 1.2968 | 0.2548 |
| 2013 | 7.2838 | <0.05 |
| 2014 | 7.3237 | <0.05 |
| 2015 | 1.8298 | 0.1762 |
| 2016 | 0.11741 | 0.7319 |
| 2017 | 8.4636 | <0.05 |
| 2018 | 5.1496 | <0.05 |
| 2019 | 1.1099 | 0.2921 |
| 2020 | 1.3958 | 0.2374 |

**S4 Table. Coefficients summary for the logistic regression models for autism diagnoses.**

| Variable | Coefficient | Std. Error | *p-value* |
| --- | --- | --- | --- |
| Model (1) |  |  |  |
| Intercept | 9.613268e-99 | 32.11009 | 2.08e-12 |
| Year | 1.119867 | 0.01594 | 1.24e-12 |
|  |  |  |  |
| Model (2) |  |  |  |
| Intercept | 1.070505e-59 | 48.47113 | 0.00509 |
| Year | 1.070729 | 0.02414 | 0.00464 |
| Gender | 1.167098 | 0.06653 | 0.02021 |

**S5 Table. Coefficients summary for the logistic regression models for Asperger’s syndrome diagnoses.**

| Variable | Coefficient | Std. Error | *p-value* |
| --- | --- | --- | --- |
| Model (1) |  |  |  |
| Intercept | 3.53067e-82 | 69.02424 | 0.00658 |
| Year | 1.097978 | 0.03428 | 0.00639 |
|  |  |  |  |
| Model (2) |  |  |  |
| Intercept | 3.602488e-52 | 77.45544 | 0.126189 |
| Year | 1.060775 | 0.03849 | 0.125279 |
| Gender | 1.376315 | 0.09080 | 0.000435 |
