# Supplementary material for: Autism trends in a medium size coastal town of England
Source: PLoS One. 2023 Jun 30;18(6):e0287808. doi: 10.1371/journal.pone.0287808 (PMC10313016; doi:10.1371/journal.pone.0287808)
Supplement: Attachment — Submitted filename: Comments.docx [file pone.0287808.s002.docx]

The article provides a valuable contribution to the understanding of autism prevalence in Fleetwood, UK. However, there are several areas where the authors could improve their work to enhance its rigor and potential impact.

Authors should number the lines of their manuscript to facilitate the review process.

Introduction: To better support the global prevalence of autism, authors may cite the recent article with DOI 10.3389/fpsyt.2023.1071181.

Authors should provide clear study objectives.

Methods: Authors should explain to what extent the population of Fleetwood represents the general population of England and whether the results of the study can be generalized to other regions.

Results: As the study only examines trends in one city, it is unclear whether the results are generalizable to other populations or geographic locations.

The study relies on diagnostic codes recorded in medical records, which may be prone to errors or inconsistencies.
