# Supplementary material for: Autism trends in a medium size coastal town of England
Source: PLoS One. 2023 Jun 30;18(6):e0287808. doi: 10.1371/journal.pone.0287808 (PMC10313016; doi:10.1371/journal.pone.0287808)
Supplement: Attachment — Submitted filename: Rebuttal.docx [file pone.0287808.s003.docx]

# Point to point replies to reviewers

We are grateful to the reviewers for their comments which helped in improving the quality of the paper and its value by making clearer the scale and generalisability of the study. We also thank the Academic Editor Prof Sebastien Kenmoe for giving us the opportunity to revise the manuscript.

Below we addressed the reviewers’ comments and suggestions. In **bold** is shown our reply to the comment while in *italic* the text as changed in the manuscript. Finally, line numbers refer to the clean version only (unmarked).

Editorial comments (EC).

[EC] Please include the following items when submitting your revised manuscript:

A rebuttal letter that responds to each point raised by the academic editor and reviewer(s). You should upload this letter as a separate file labeled 'Response to Reviewers'.

A marked-up copy of your manuscript that highlights changes made to the original version. You should upload this as a separate file labeled 'Revised Manuscript with Track Changes'.

An unmarked version of your revised paper without tracked changes. You should upload this as a separate file labeled 'Manuscript'.

**All files are uploaded.**

[EC] If applicable, we recommend that you deposit your laboratory protocols in protocols.io to enhance the reproducibility of your results. Protocols.io assigns your protocol its own identifier (DOI) so that it can be cited independently in the future. For instructions see: https://journals.plos.org/plosone/s/submission-guidelines#loc-laboratory-protocols. Additionally, PLOS ONE offers an option for publishing peer-reviewed Lab Protocol articles, which describe protocols hosted on protocols.io. Read more information on sharing protocols at https://plos.org/protocols?utm_medium=editorial-email&utm_source=authorletters&utm_campaign=protocols.

**Not applicable.**

[EC] Please ensure that your manuscript meets PLOS ONE's style requirements, including those for file naming. The PLOS ONE style templates can be found at <https://journals.plos.org/plosone/s/file?id=wjVg/PLOSOne_formatting_sample_main_body.pdf> and <https://journals.plos.org/plosone/s/file?id=ba62/PLOSOne_formatting_sample_title_authors_affiliations.pdf>

**Corrections made, including list of supplementary files (currently collapsed in a single document).** Page: 15.

[EC] Please ensure that you have specified (1) whether consent was informed and (2) what type you obtained (for instance, written or verbal, and if verbal, how it was documented and witnessed). If your study included minors, state whether you obtained consent from parents or guardians. If the need for consent was waived by the ethics committee, please include this information.

**We have clarified this point in the Ethics section of the article. We added:** ‘*Anonymised data was shared by each Fleetwood GP practice only considering patients that opted in the option to consent their data to be used anonymised for research purposes*’. Lines: 139-141.

[EC] We note that the grant information you provided in the ‘Funding Information’ and ‘Financial Disclosure’ sections do not match. When you resubmit, please ensure that you provide the correct grant numbers for the awards you received for your study in the ‘Funding Information’ section.

**We have added the funder which comes from an EPSRC IAA Funding Programme. This funding does not have a grant number. However, we were not able to change the financial disclosure statement since not available in the revision tabs.**

[EC] In your Data Availability statement, you have not specified where the minimal data set underlying the results described in your manuscript can be found. PLOS defines a study's minimal data set as the underlying data used to reach the conclusions drawn in the manuscript and any additional data required to replicate the reported study findings in their entirety. All PLOS journals require that the minimal data set be made fully available. For more information about our data policy, please see <http://journals.plos.org/plosone/s/data-availability>. Upon re-submitting your revised manuscript, please upload your study’s minimal underlying data set as either Supporting Information files or to a stable, public repository and include the relevant URLs, DOIs, or accession numbers within your revised cover letter. For a list of acceptable repositories, please see http://journals.plos.org/plosone/s/data-availability#loc-recommended-repositories. Any potentially identifying patient information must be fully anonymized. Important: If there are ethical or legal restrictions to sharing your data publicly, please explain these restrictions in detail. Please see our guidelines for more information on what we consider unacceptable restrictions to publicly sharing data: http://journals.plos.org/plosone/s/data-availability#loc-unacceptable-data-access-restrictions. Note that it is not acceptable for the authors to be the sole named individuals responsible for ensuring data access.We will update your Data Availability statement to reflect the information you provide in your cover letter. We note that you have stated that you will provide repository information for your data at acceptance. Should your manuscript be accepted for publication, we will hold it until you provide the relevant accession numbers or DOIs necessary to access your data. If you wish to make changes to your Data Availability statement, please describe these changes in your cover letter and we will update your Data Availability statement to reflect the information you provide.

**The data used contain: age, sex and diagnosis for 239 patients in Fleetwood. Fleetwood is a small community (around 30,000 population) therefore releasing this data may make some individuals identifiable even if the data is anonymised. We therefore decided to make the data available upon request with the following statement:** ‘*Data available upon request to the IT/DATA Quality Lancashire of NHS Midlands and Lancashire Commissioning Support Unit corresponding Louise Ross (louise.ross13@nhs.net) and only after ethical approval is granted by the requesting institution*’. **However, we are happy to share the data with editors and reviewers if the wish to look at them.**

[EC] While revising your submission, please upload your figure files to the Preflight Analysis and Conversion Engine (PACE) digital diagnostic tool, https://pacev2.apexcovantage.com/. PACE helps ensure that figures meet PLOS requirements. To use PACE, you must first register as a user. Registration is free. Then, login and navigate to the UPLOAD tab, where you will find detailed instructions on how to use the tool. If you encounter any issues or have any questions when using PACE, please email PLOS at figures@plos.org. Please note that Supporting Information files do not need this step.

**Done.**

[EC] Please include captions for your Supporting Information files at the end of your manuscript, and update any in-text citations to match accordingly. Please see our Supporting Information guidelines for more information: <http://journals.plos.org/plosone/s/supporting-information>.

**Corrections made.**

Reviewer 1 [R1]

[R1] The paper presents clear but minimal data about the prevalence and incidence of autism diagnoses from primary care health records from an economically deprived region in England. The major findings are that the prevalence and incidence are lower than expected from other reports and the rates increased in the last 10 years. However, the paper is limited by little information about the families or demographics of the sample other than the gender of the children. The numbers are also quite small so claims of a less pronounced sex difference could be due to the very small numbers of patients, particularly females. The paper is well-written and clear; the limitations are primarily the lack of other information.

**We agree with the reviewer on the major limitations of our analysis and therefore on the generalisability of our results. However, the study highlights the lack of studies and quantitative assessment around the gender disparity issue. We aim to inspire more research on this topic and to evaluate the effect of temporal trends in the incidence of ASD. Finally, we provide an example of research aimed at local public health interventions where patterns and trends are the essential information for developing such initiatives. We have changed several sections to reflect these issues.**

**We added this sentence at the end of the abstract:** *‘However, due to the small sample size of the study, confirmation of the gender results and identification of the factors determining the temporal trends are needed in order to determine the gender effects in ASD diagnosis.’* Lines: 38-40.

**And one at the end of the Introduction**: ‘*Here we present a study that aims to investigate the trends in ASD diagnoses in Fleetwood as typical English deprived coastal community. While findings are not generalisable at regional and national level, they can still provide key information in terms of at-risk groups and trends over the last two decades for public health interventions.*’ Lines: 106-109.

**And finally in the discussions**: ‘*Firstly, the small sample size (a population size of around 30,000 people) and the specific conditions of Fleetwood (in terms of deprivation and other health and socio-economic measures) do not allow generalisation of the results – i.e. to regional or national levels. In addition, having considered only two classes representing a complex plethora of disorders could have reduced the capacity to discriminate between the true drivers of trends and gender spectrum ratios. Most importantly, due to identifiability restrictions it was not possible to associated other demographic and environmental factors that may be related to ASD diagnoses (genetic, environmental etc…) [30-34].*’ Lines: 244-252.

**We thank the reviewer for this valuable comment.**

Reviewer 2 [R2]

[R2] The article provides a valuable contribution to the understanding of autism prevalence in Fleetwood, UK. However, there are several areas where the authors could improve their work to enhance its rigor and potential impact.

**We thank the reviewer for this positive comment.**

[R2] Authors should number the lines of their manuscript to facilitate the review process.

**We have done this correction and apologise for having missed it in the previous version.**

[R2] Introduction: To better support the global prevalence of autism, authors may cite the recent article with DOI 10.3389/fpsyt.2023.1071181.

**Thank you. We have cited this article in the following sentence of the Introduction:** ‘*From a recent systematic review and meta-analysis, the global prevalence of ASD was estimated to be 0.72% [3].*’ Lines: 71-72.

[R2] Authors should provide clear study objectives.

We have included an aim statement at the end of the Introduction: *Here we present a study that aims to investigate the trends in ASD diagnoses in Fleetwood as typical English deprived coastal community. While findings are not generalisable at regional and national level, they can still provide key information in terms of at-risk groups and trends over the last two decades for public health interventions.*’ Lines: 106-109.

[R2] Methods: Authors should explain to what extent the population of Fleetwood represents the general population of England and whether the results of the study can be generalized to other regions. Results: As the study only examines trends in one city, it is unclear whether the results are generalizable to other populations or geographic locations.

**This is an important point and similar to the comment from reviewer [1]. As stated above, we agree with this issue. Please see above the reply to Reviewer 1 and the changes made to the article in order to clarify this point.**

[R2] The study relies on diagnostic codes recorded in medical records, which may be prone to errors or inconsistencies.

Thanks, we have now added a sentence in the Discussions to acknowledge this potential issue: ‘*Finally, it is important also to recognise that when using diagnostic codes to identify patients, there is always a risk that the coding is inconsistent between users, or, that there have been errors in the medical records.*’ Lines: 254-256.
